# Sensitization of radio-resistant prostate cancer cells with a unique cytolethal distending toxin

**DOI:** 10.18632/oncotarget.2133

**Published:** 2014-06-26

**Authors:** Chih-Ho Lai, Chia-Shuo Chang, Hsin-Ho Liu, Yuh-Shyan Tsai, Feng-Ming Hsu, Yung-Luen Yu, Cheng-Kuo Lai, Leah Gandee, Rey-Chen Pong, Heng-Wei Hsu, Lan Yu, Debabrata Saha, Jer-Tsong Hsieh

**Affiliations:** ^1^ Department of Urology, University of Texas Southwestern Medical Center, Dallas, Texas, USA; ^2^ School of Medicine, China Medical University, Taichung, Taiwan; ^3^ Department of Urology, Medical College and Hospital, National Cheng Kung University, Tainan, Taiwan; ^4^ Department of Oncology, National Taiwan University Hospital, Taipei, Taiwan; ^5^ Department of Radiation Oncology, University of Texas Southwestern Medical Center, Dallas, Texas, USA; ^6^ Graduate Institute of Cancer Biology, China Medical University, Taichung, Taiwan

**Keywords:** prostate cancer, ionizing radiation, radio-resistance, cytolethal distending toxin

## Abstract

Cytolethal distending toxin (CDT) produced by *Campylobacter jejuni* is a genotoxin that induces cell-cycle arrest and apoptosis in mammalian cells. Recent studies have demonstrated that prostate cancer (PCa) cells can acquire radio-resistance when DOC-2/DAB2 interactive protein (DAB2IP) is downregulated. In this study, we showed that CDT could induce cell death in DAB2IP-deficient PCa cells. A combination of CDT and radiotherapy significantly elicited cell death in DAB2IP-deficient PCa cells by inhibiting the repair of ionizing radiation (IR)-induced DNA double-strand break (DSB) during G2/M arrest, which is triggered by ataxia telangiectasia mutated (ATM)-dependent DNA damage checkpoint responses. We also found that CDT administration significantly increased the efficacy of radiotherapy in a xenograft mouse model. These results indicate that CDT can be a potent therapeutic agent for radio-resistant PCa.

## INTRODUCTION

Prostate cancer (PCa) is one of the most prevalent male cancers in many western developed countries, and its incidence is increasing worldwide [1]. For patients with inoperable disease or high risk of cancer recurrence, radiation therapy is considered an optimal choice [2, 3]. However, some of these patients present with intrinsic radio-resistance or develop radio-resistance prior to or after radiotherapy [4]. Although recent advances in radiotherapy have improved the disease-free survival rates of patients with disseminated PCa, disease recurrence remains inevitable [5, 6].

DOC-2/DAB2 interactive protein (DAB2IP) is a potent tumor suppressor in PCa, modulating several pathways including that of mitogen-activated protein kinase and phosphoinositide 3-kinase/protein kinase B (Akt) [7]. Loss of DAB2IP expression in PCa cells has been reported to provide a survival advantage and resistance to stress-induced apoptosis [8]. Reduced expression of DAB2IP in PCa cells also induces epithelial-mesenchymal transition (EMT), leading to cancer metastasis [9], and these cells acquire radio-resistance to ionizing radiation (IR)-induced apoptosis [10]. Therefore, it is imperative to develop a strategy to overcome radio-resistant phenotype of these cells in order to maximize the efficacy of radiation therapy.

Cytolethal distending toxin (CDT) is a bacterial genotoxin [11]. It is a tripartite protein toxin composed of the 3 subunits, CdtA, CdtB, and CdtC [12], that are encoded by an operon comprising *cdtA*, *cdtB*, and *cdtC* [13]. In a holotoxin containing all the three subunits, CdtA and CdtC are to interact with the cell membrane and enable the translocation of CdtB across the cell membrane [14]. Cellular cholesterol plays an important role in mediating CDT binding to the cell membrane, where it serves as a portal for CdtB delivery into host cells and induction of cell intoxication [15-18]. In addition, the nuclear-translocated CdtB exhibits type I deoxyribonuclease activity, which causes DNA damage leading to cell-cycle arrest at G2/M [11]. Several studies have shown some promising therapeutic effect of CDT, derived from other bacterial species, on oral cancer cells [19-21]. Thus, we decided to explore the effect of CDT on radio-resistant PCa cells. Data from this study indeed reveal that CDT can overcome radio-resistance of PCa cells by intervening the repair of the radiation-induced DNA double-strand break (DSB) and ataxia telangiectasia mutated (ATM)-dependent DNA damage checkpoint responses. Taken together, CDT in combination with radiotherapy can be considered a unique therapeutic strategy to aggressive PCa.

## RESULTS

### CDT induces cell death in DAB2IP- knockdown (KD) cells

**Figure 1:**
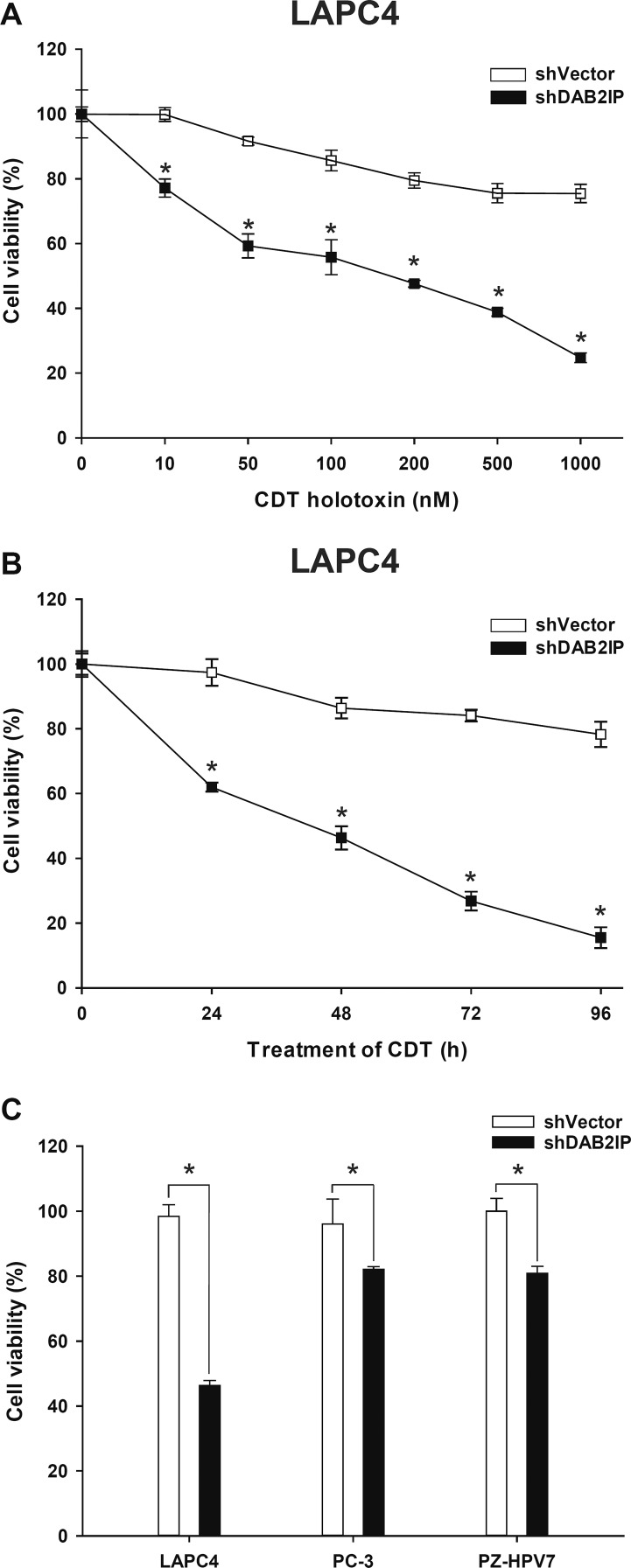
DAB2IP-deficient PCa cells are susceptible to CDT Cell viability of LAPC4 shVector and shDAB2IP cells in response to CDT treatment at different concentrations and incubation times: (A) CDT at different concentrations ranging from 0 to 1000 nM was added to cells for 24 h, and (B) cells were exposed to 200 nM CDT for the indicated incubation time. (C) Cytotoxicity of CDT in shVector and shDAB2IP PCa cells (LPAC4, PC-3, and PZ-HPV-7) determined by MTT assay. The asterisk (*) indicates statistical significance (*P* < 0.05) determined by Student's *t*-test.

Our recent data demonstrated that loss of DAB2IP in PCa cells elicited epithelial-to-mesenchymal transition (EMT), leading to cancer metastases [8] and radio-resistance [9]. Considering the therapeutic potential of CDT, we aimed to evaluate whether CDT holotoxin exhibits any cytolethal activity in PCa cells. Three cell lines, LAPC4, PC-3, and PZ-HPV-7, were included in this study. Western blot analysis showed that DAB2IP levels were significantly reduced in all three DAB2IP-KD (i.e., shDAB2IP) lines compared to their corresponding controls (i.e., shVector) (Supplementary Figure S1). LAPC4 shVector and shDAB2IP cells were treated with different concentrations (0–1000 nM) of CDT for varying incubation times (0–96 h), and CDT cytotoxicity was determined. As shown in Figure 1A and B, CDT potently induced cell death in LAPC4 shDAB2IP cells in a concentration- and time-dependent manner with an IC_50_ of approximately 200 nM (Figure 1A). To further evaluate whether CDT exhibited cytolethal activity in these cells, we treated cells with CDT at 200 nM for 24 h. Cell distention was apparent in shDAB2IP cells treated with CDT (Supplementary Figure S2). Similarly, a significant cytotoxic effect of CDT treatment was observed in DAB2IP-deficient cells (Figure 1C), whereas this was not observed in control cells.

### CDT increases the sensitivity of DAB2IP-deficient PCa cells to IR

**Figure 2:**
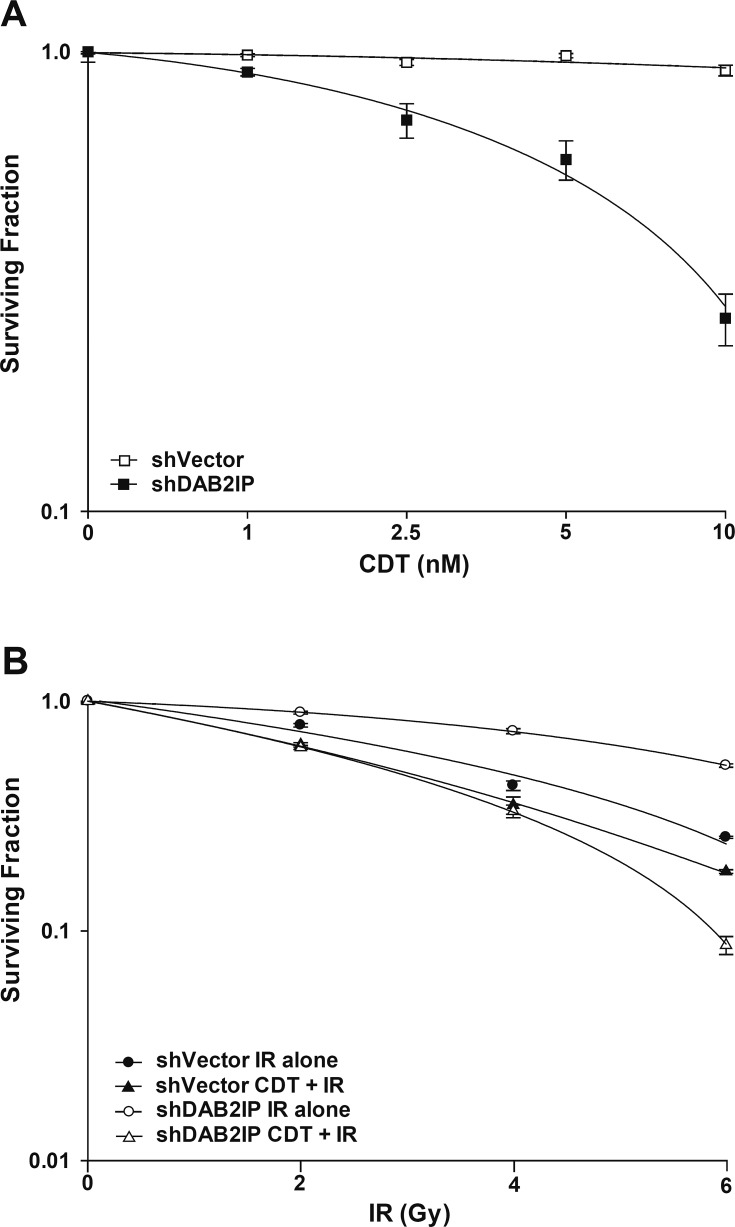
CDT enhances radio-sensitivity in DAB2IP-deficient PCa cells (A) CDT, at the indicated concentration, was added to shVector or shDAB2IP cells before IR. (B) shVector or shDAB2IP cells were treated with IR (2–6 Gy) alone, or CDT (10 nM) combined with IR. After 10 days of incubation, colonies were stained with crystal violet and survival was calculated using clonogenic assays.

We then assessed whether CDT enhances the sensitivity of PCa cells to IR. Cells were first treated with CDT alone (0–10 nM), and viability was determined using clonogenic assay. We found that shDAB2IP cells were more susceptible to CDT than shVector cells, and the effect of CDT was dose-dependent (Figure 2A). In addition, our data also demonstrated that shDAB2IP cells were more susceptible to combined treatment than IR alone (Figure 2B). The surviving fraction (SF) in response to IR at 2 Gy (SF2) for shVector and shDAB2IP cells was 0.75 and 0.87, respectively. Notably, when cells were treated with a combination of CDT and IR, the SF2 significantly decreased to 0.68 for shVector cells and 0.66 for shDAB2IP cells. The dose enhancement ratio (DER) at 10% survival was 1.12 for shVector cells and 2.05 for shDAB2IP cells. SFs at different IR doses and the corresponding DERs are shown in Table 1. These results demonstrate that CDT synergistically enhanced the effects of IR on shDAB2IP cells but not in control shVector cells.

**Table 1:** Radiation dose enhancement ratios for cytolethal distending toxin

shVector				shDAB2IP			
SF	IR (Gy)	CDT+IR	DER	SF	IR (Gy)	CDT+IR	DER
0.1	7.00	6.23	1.12	0.1	11.74	5.71	2.05
0.2	6.22	5.51	1.13	0.2	10.47	5.06	2.07
0.3	5.45	4.78	1.14	0.3	9.20	4.40	2.09
0.4	4.68	4.05	1.15	0.4	7.93	3.74	2.12
0.5	3.91	3.33	1.17	0.5	6.67	3.08	2.16
0.6	3.14	2.60	1.21	0.6	5.40	2.42	2.23

SF: surviving fraction; IR; ionizing radiation; CDT: cytolethal distending toxin; DER: dose enhancement ratio.

### CDT with radiation results in synergistic anti-tumor activity in DAB2IP-deficient PCa cells

**Figure 3:**
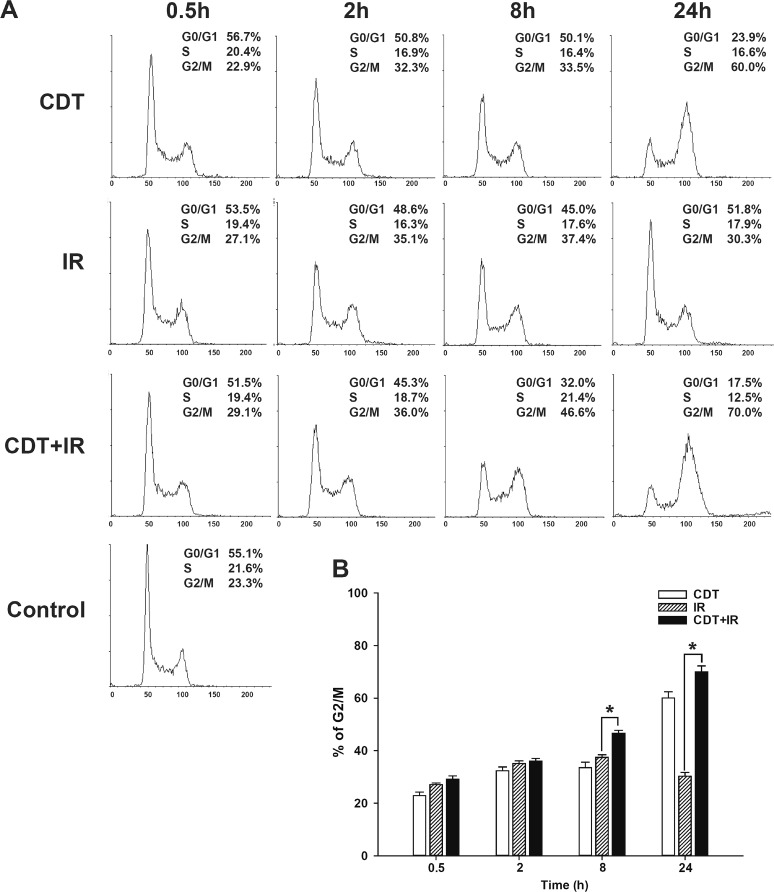
CDT enhanced cell cycle arrest at G2/M in radio-resistant DAB2IP-deficient PCa cells (A) LAPC4 shDAB2IP cells were untreated (control), exposed to CDT (10 nM) alone, IR (1 Gy) alone, or CDT for 1 h prior to IR treatment (CDT+IR). Cells were then collected at various time points as indicated. Cell cycle distribution was based on DNA content analyzed by flow cytometry. The proportions of cells in G0/G1, S, and G2/M phases are indicated on the right of each histogram. (B) The percentage of cells in G2/M was calculated and plotted as intensity bars. The asterisk (*) indicates statistical significance (*P* < 0.05) determined by Student's *t*-test.

CDT acts by eliciting cell cycle arrest at G2/M, subsequently inducing apoptosis [22]. To evaluate the effect of CDT on cell cycle arrest in shDAB2IP cells with or without IR, LAPC4 shDAB2IP cells were treated with CDT alone, IR alone, and CDT combined with IR. Cells were then harvested at various time points (0.5–24 h) after treatment and subjected to cell cycle analysis. Our data showed that when treated with CDT alone, the number of LAPC4 shDAB2IP cells arrested in G2/M phase gradually increased from 0.5 to 24 h (Figure 3A). IR alone also induced a time-dependent G2/M arrest during 0.5–8 h of incubation time, but such an effect was attenuated at 24 h. In contrast, a combination treatment with CDT and IR led to a significant synergistic G2/M cell cycle arrest observed at 24 h. These results suggest that CDT-induced cell death in radio-resistant PCa cells may be mediated through G2/M cell cycle arrest.

**Figure 4:**
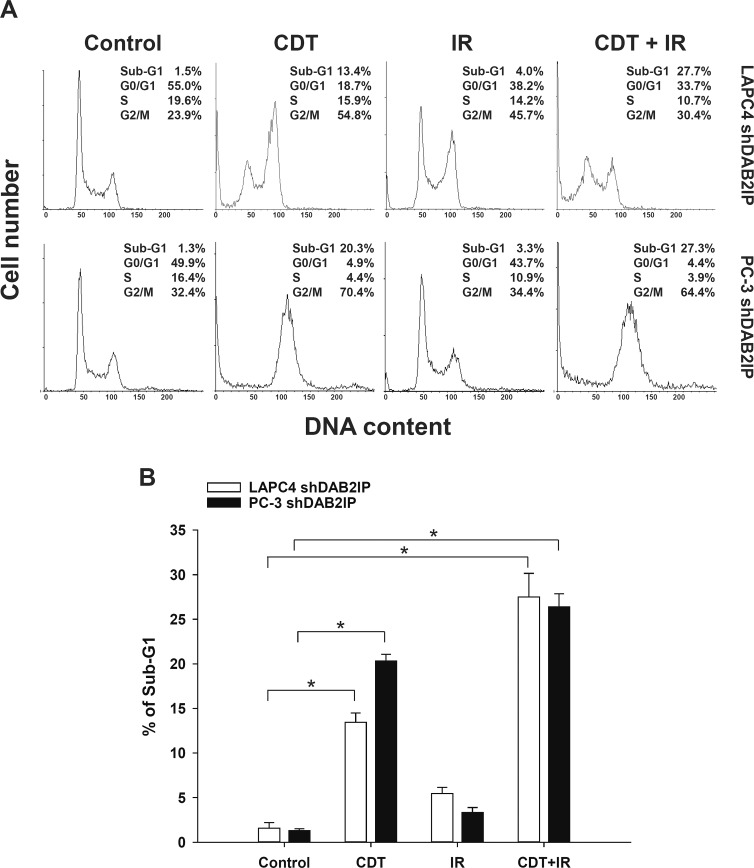
Prolonged exposure to CDT induced apoptosis in radio-resistant DAB2IP-deficient PCa cells (A) LAPC4 shDAB2IP and PC3 shDAB2IP cells were untreated (control) or treated with CDT (50 nM) alone, IR (2 Gy) alone, and a combination of CDT and IR. Cells were then incubated for 48 h. Cell cycle distribution was based on DNA content analyzed by flow cytometry. The percentages of cells in sub-G1, G0/G1, S, and G2/M phases are indicated on the right of each histogram. (B) The percentages of cells in sub-G1 were calculated and plotted as intensity bars. The asterisk (*) indicates statistical significance (*P* < 0.05) determined by Student's *t*-test.

We further investigated whether CDT induces cell death in LAPC4 shDAB2IP and PC-3 shDAB2IP cells. After treatment with CDT or IR, cells were incubated for 48 h, and cell cycle analysis was performed by flow cytometry. As shown in Figure 4A, the proportion of sub-G1 cells treated with IR alone was similar to that in the untreated group. CDT treatment, however, significantly increased the proportion of sub-G1 cells. In addition, a combination of CDT and IR synergistically increased the sub-G1 population in shDAB2IP PCa cells after 48 h (Figure 4). These results indicate that CDT can effectively increase IR-induced apoptosis in DAB2IP-deficient cells.

### Mechanism underlying the synergistic enhancement of radio-sensitivity in PCa cells by CDT

**Figure 5:**
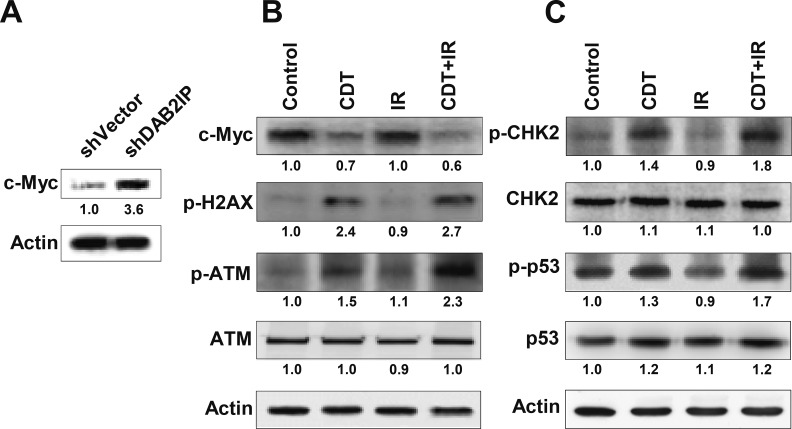
CDT increases IR-induced DSB in DAB2IP-deficient PCa cells The expression levels of phosphorylated proteins (γ-H2AX, ATM, CHK2, and p53) and c-Myc in untreated (control) shDAB2IP-KD cells and those treated with CDT (CDT), irradiation (IR), and CDT combined with radiation (CDT+IR) for 24 h are shown. Actin expression is used as the loading control. The expression level of each protein is quantified by signal intensity and is indicated at the bottom of each lane.

A recent study has suggested that c-Myc expression is required to activate ATM-dependent DNA damage checkpoint responses when cells are exposed to IR or CDT [23]. We therefore tested whether c-Myc plays a critical role in the CDT-enhanced radio-sensitivity of DAB2IP-deficient PCa cells. As shown in Figure 5A, c-Myc expression was highly elevated in LAPC4 shDAB2IP cells compared to controls. Although c-Myc expression levels remained unchanged in cells treated with IR alone compared to untreated cells (Figure 5B), they significantly decreased when cells were treated with CDT or a combination of CDT and IR. To further demonstrate the role of c-Myc, shVector cells were transiently transfected with c-Myc expression vector followed by treatment with CDT for 24 h. An increase in CDT-induced cell death was observed in c-Myc overexpression cells compared to mock-transfected cells (Supplementary Figure S3). These results suggest that c-Myc levels are critical for CDT-induced cell death. We subsequently profiled the status of other key molecules associated with DNA DSB, and the results indicate that the expression of phosphorylated γ-H2AX and ATM increased in cells treated with either CDT or a combination of CDT with IR, consistent with increased levels of phosphorylated CHK2 and p53 (Figure 5B and 5C).

### CDT enhancement of radio-sensitivity in DAB2IP-deficient PCa cells is associated with compromised DSB repair

**Figure 6:**
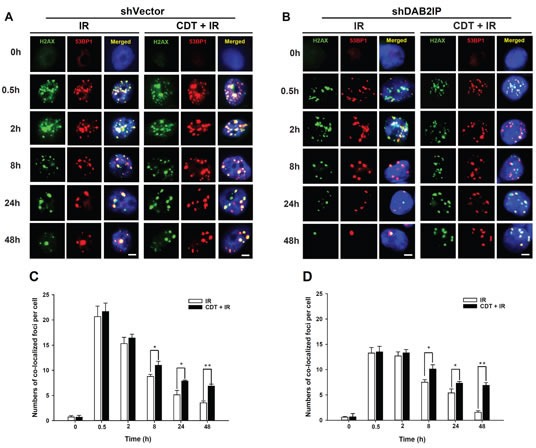
CDT enhances IR-induced DSB in DAB2IP-deficient PCa cells (A) shVector or (B) shDAB2IP cells were untreated (0 h), exposed to IR (2 Gy) alone, or treated with a combination of CDT (50 nM) and IR (2 Gy) for the indicated time and immunostained for phospho-γ-H2AX (green) and 53BP1 (red) foci. The number of co-localized foci (phospho-γ-H2AX and 53BP1) was determined for each time point in (C) shVector and (D) shDAB2IP cells. The remaining merged foci in the nuclei were counted in 3 independent experiments (50 nuclei each). Statistical significance was evaluated using Student's *t-*test (*, *P* < 0.05; **, *P* < 0.01).

CdtB is known to exhibit endonuclease activity to elicit DNA damage signaling (i.e., γ-H2AX expression) in response to G2/M cell cycle arrest [24]. The recruitment of both phospho-γ-H2AX and 53BP1 at the damage sites often indicates an early step in response to DSB. We therefore examined CDT-induced cell death in PCa cells by analyzing focus formation of phospho-γ-H2AX and 53BP1 [10]. LAPC4 cells (shVector or shDAB2IP) were treated with CDT (50 nM) for varying amounts of time and subjected to immunofluorescence staining for phospho-γ-H2AX (green) and 53BP1 (red) foci. As shown in Figure 6, the co-localization of γ-H2AX and 53BP1 foci significantly increased in shVector and shDAB2IP cells upon exposure to IR for 0.5 h compared to untreated cells (0 h). However, the remaining foci were almost abolished in shDAB2IP cells treated with IR at 48 h. Nevertheless, in cells treated with a combination of CDT and IR, the high level of foci formation remained observable in shDAB2IP PCa cells at 48 h (Figure 6B and D). These results indicate that CDT enhances IR-induced DSB in DAB2IP-deficient PCa cells.

### Effect of CDT, IR, and a combination of CDT and IR on PCa growth

**Figure 7:**
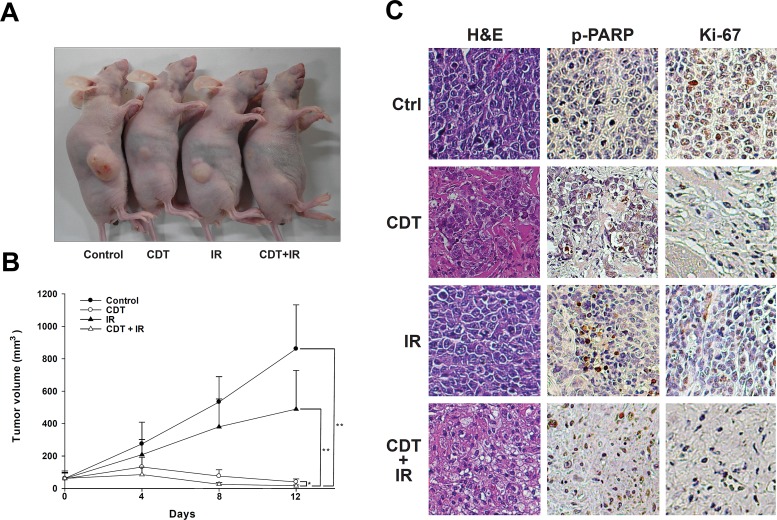
CDT synergistically enhanced IR-attenuated PCa growth in a mouse xenograft model A total of 36 mice were divided into 4 experimental groups: control (n = 9), CDT (n = 9), IR (n = 9), and CDT combined with IR (n = 9). LAPC4 shDAB2IP cells were inoculated into nude mice as described. Treatments were administered on days 0, 4, and 8. (A) Macroscopic images of LAPC4 shDAB2IP xenograft tumors treated with vehicle control, CDT alone, IR alone, and CDT combined with IR are shown. (B) The tumor volume was calculated; data were expressed as the mean ± standard deviation and evaluated using two-way analysis of variance (*, *P* < 0.05; **, *P* < 0.01). (C) Representative hematoxylin-eosin and immunohistochemistry staining of tumor samples is shown. For immunohistochemistry, paraffin sections were stained with specific antibodies against cleaved PARP and Ki-67 (magnification: 400×).

To further demonstrate the role of CDT as a radio-sensitizer in radiotherapy for PCa, we evaluated pre-clinical experimental therapy using a xenograft animal model. Tumors were treated with CDT (2.5 mg/kg). For combination therapy, CDT was administered 6 h before radiation. Fractionated radiation (4 Gy × 3) was administered on days 0, 4, and 8. Our results showed that CDT alone was able to significantly reduce tumor growth. Nevertheless, combination therapy showed the best efficacy compared to controls (*P* < 0.01), IR alone (*P* < 0.01), and CDT alone (*P* < 0.05) (Figure 7). Our data demonstrate that CDT can effectively inhibit the growth of radio-resistant PCa tumors *in vivo*.

In comparison to controls, tumors treated with a combination of CDT and IR clearly showed the presence of apoptotic cells determined by TUNEL. TUNEL-stained positive cells were not observed in samples from the control and IR-alone groups (Supplementary Figure S4). Additionally, immunohistochemical analyses revealed that the cell proliferation marker (Ki-67) was diminished and the apoptotic marker (p-PARP) increased in tumors treated with a combination of CDT and IR (Figure 7C), indicating that CDT increased IR-induced cell death via apoptosis. Taken together, the results of this pre-clinical study indicate that CDT could be a potent therapeutic agent for PCa and can be provided in combination with radiotherapy.

## DISCUSSION

Bacterial toxins have been extensively studied for their biomedical applications in cancer therapy. For example, Shiga toxin, produced by *Shigella dysenteriae* and enterohemorrhagic *Escherichia coli*, has been found to inhibit tumor growth in mouse xenograft models [25]. Diphtheria toxin inhibits protein synthesis, resulting in cancer cell apoptosis [26]. In addition, *Clostridium perfringens* enterotoxin displays acute cytotoxic activity and induces tumor necrosis *in vivo* [27]. These findings have strongly supported the use of bacterial toxins as potential cancer therapeutic agents.

DAB2IP is a novel member of the Ras-GTPase-activating protein family that plays an important role in regulating cell proliferation, survival, and apoptosis by inactivating PI3K-Akt via apoptosis signal-regulating kinase 1 (ASK1) activation pathways [8, 9]. Downregulation of DAB2IP in PCa cells renders these cells resistant to stress-induced apoptosis [8], radiotherapy [10], and chemotherapy [28]. Loss of DAB2IP expression is frequently detected in PCa cells and is associated with an increased risk for tumor metastasis [9, 29]. Furthermore, a single-nucleotide polymorphism probe (rs1571801) in the *DAB2IP* gene has been nominally associated with aggressive PCa phenotypes [30-33]. In addition, the DAB2IP expression level determined by immunohistochemical staining of needle biopsy specimens from PCa patients has been shown to be associated with recurrence-free survival in a retrospective cohort analysis [34]. These findings suggest that DAB2IP is a prognostic marker for aggressive PCa.

We have previously shown that loss of DAB2IP can increase EMT in PCa, leading to cancer metastases [9] and radio-resistance [10]. A decrease in DAB2IP expression in PCa cells has been reported to induce accelerated DSB repair, a G2/M checkpoint response and a means of escaping apoptosis [10]. On the basis of the above-mentioned clinical evidence and DAB2IP mechanism of action, a targeted therapeutic strategy for DAB2IP-deficient PCa could potentially be developed. In this study, we found that CDT, a bacterial genotoxin secreted by *C. jejuni*, can induce cell cycle arrest at G2/M and apoptosis in DAB2IP-deficient PCa cells exhibiting a radio-resistant phenotype. Such findings suggest that CDT can sensitize these cells to IR. Our data clearly demonstrate that CDT, when combined with IR, has a synergistic cytotoxic effect. The mechanisms underlying radio-sensitization induced by CDT in DAB2IP-deficient PCa cells are attributed to the degradation of host cell DNA and cell cycle arrest, resulting in an increased induction of apoptosis.

Both IR and CDT have been reported to exhibit similar cytotoxic effects: cell cycle arrest and increased induction of apoptosis [14, 35]. Our results show that CDT dramatically induced cell death in shDAB2IP cells but not in shVector cells (Figures 1 and 2). When treated with a combination of CDT and IR, shDAB2IP cells demonstrated a different cell cycle pattern from that when treated with IR alone at 24 h. We noticed a prolonged G2/M arrest followed by apoptosis in cells treated with a combination of CDT and IR (Figures 3 and 4). In contrast, a significant sub-G1 population was not observed in shDAB2IP cells treated with IR alone (Figure 4). This result indicates that CDT enhancement of IR-induced cell death was mediated through cell cycle arrest followed by activation of the apoptotic pathway.

Activation of checkpoint responses to genotoxic stress is a major factor in the prevention of carcinogenesis [36]. Cells exposed to DNA damaging agents often undergo cell cycle arrest followed by senescence or eventually apoptosis [37]. c-Myc has been reported to play an important role in regulating cancer cell growth, differentiation, apoptosis, and metabolism [38]. In this study, an increase in c-Myc expression was observed in shDAB2IP cells compared to shVector cells (Figure 5A). After shDAB2IP cells were exposed to CDT or a combination of CDT and IR, their c-Myc expression declined, but the expressions of phosphoproteins such as γ-H2AX, ATM, CHK2, and p53 were significantly elevated (Figures 5B and C). These results are consistent with a previous study reporting that c-Myc can enhance the DNA damage response by stimulating ATM and CHK2 phosphorylation, resulting in cell death [23]. Although our data clearly indicate that CDT can target c-Myc, which is critical for PCa growth and survival, the exact mechanism requires further study.

Although γ-H2AX can recruit DNA repair proteins nearby DNA damage site and the γ-H2AX-foci containing p53 binding protein 1 (53BP1) [39], γ-H2AX is not always associated with DNA damage. Previous studies have demonstrated that γ-H2AX expression may be elevated in senescent cells, thus leading to activation of the DNA damage response (DDR) in the absence of DNA damage [40, 41]. Additionally, the pseudo-DDR, or the DDR in senescent cells, is represented by γ-H2AX phosphorylation without detectable DNA damage [42]. To strengthen our finding that CDT-induced cell death was mediated by DNA DSB, we performed additional studies involving comet assays. As shown in Supplementary Figure S5, the comets were significantly increased in all 3 shDAB2IP cell lines (LAPC4, PC-3, and PZ-HPV-7) following treatment with CDT relative to the comets in untreated cells. In contrast, CDT only slightly increased the comets in the shVector cells. These results are consistent with the phosphorylated γ-H2AX expression data (Figures 5 and 6), indicating that CDT-induced cell death in DAB2IP-deficient prostate cancer cells was mediated by DSB.

Alteration of ATM expression has been reported to play an important role in prostate tumorigenesis [43]. Moreover, a DNA DSB in eukaryotic cells is generally accompanied by the formation of hundreds of histone γ-H2AX molecules in the chromatin flanking the break site [44]. Our data show that either CDT or CDT combined with IR induced ATM-dependent checkpoint responses (Figure 5). However, when the combination treatment was applied, the ATM–CHK2–p53 apoptotic pathway was dramatically activated, which is thought to be a p53-dependent apoptosis response to genotoxic stress [45]. These findings indicate that CDT-enhanced radio-sensitivity in DAB2IP-deficient cells is associated with compromised DSB repair.

Previous studies have indicated that several anti-cancer compounds, including honokiol [46], paclitaxel (Taxol) [47], gemcitabine [48], and epothilone B [49], can enhance the sensitivity of tumors to radiotherapy. However, significant side effects associated with these anti-cancer drugs are also frequently reported [50-52]. Previous investigations have utilized CDT from *Aggregatibacter actinomycetemcomitans* for the treatment of human gingival squamous carcinoma and oral cancer cells [19-21]. Recently, our group developed chitosan/heparin nanoparticles for the delivery of *C. jejuni* CdtB as a potential therapeutic agent for gastric cancer [53]. Our *in vivo* xenograft experiments in this study further demonstrated that *C. jejuni* CDT effectively inhibited the growth of radio-resistant PCa (Figure 7). Considering the biological safety and low CDT concentration administered in cancer therapy, our study supports the utilization of CDT in therapy for radio-resistant PCa, particularly in cases with loss of DAB2IP expression.

In conclusion, our findings indicate that CDT can enhance the effect of IR in radio-resistant PCa. The molecular mechanism of such radio-sensitization might be attributable to the attenuation of DSB repair and the long-term cell cycle arrest at G2/M followed by the induction of the apoptotic pathway. We believe CDT can be utilized as a potential personalized medicine in combination with radiotherapy to target PCa with DAB2IP deficiency.

## MATERIALS AND METHODS

### Antibodies and reagents

Antibody specific for phospho-p53 was purchased from Santa Cruz Biotechnology (Santa Cruz, CA). Poly (ADP-Ribose) P polymerase (PARP) antibody was purchased from BioLegend (San Diego, CA). Antibodies against cleaved PARP (Asp214), phospho-ATM and phospho-checkpoint kinase 2 (CHK2) were purchased from Cell Signaling (Danvers, MA). Anti-phospho-γ-H2AX (Ser139) antibody was purchased from Millipore (Billerica, MA). Alexa Fluor 488–conjugated goat anti-mouse IgG, and Alexa Fluor 594–conjugated goat anti-rabbit IgG, and 4’,6-diamidino-2-phenylindole (DAPI) were purchased from Molecular Probes (Invitrogen, Carlsbad, CA). All other reagents were obtained from Sigma-Aldrich (St. Louis, MO).

### Cell culture

The shRNA system (pGIPZ-lentiviral-shRNAmir from Open Biosystems, Huntsville, AL) was used to knockdown (KD) endogenous DAB2IP in various prostate epithelial cell lines: control (shVector) and knockdown (shDAB2IP) cells were selected under puromycin. PC-3 cell line is maintained in RPMI1640 supplemented with 5% fetal bovine serum (FBS) (Hyclone, Logan, UT) in a humidified atmosphere containing 5% CO_2_ [9]. LAPC4 is maintained in Iscove's Modified Dulbecco's Medium (IMDM) (Gibco, Grand Island, NY) supplemented with 5% FBS. PZ-HPV-7, an immortalized cell line derived from the peripheral zone of a benign prostate, is maintained in prostate epithelial growth medium (PrEGM) (Lonza, Basel, Switzerland) with 5% FBS.

### Purification of recombinant CDT proteins

Recombinant His-tagged CDT subunits were cloned by following the standard protocols as described previously [17]. The expressed His-tagged CdtA, CdtB, and CdtC fusion proteins were purified by metal affinity chromatography (Clontech, Palo-Alto, CA) and assessed by SDS-PAGE. Each purified protein was subjected to ToxinEraser (GenScript, Piscataway, NJ) for removing of endotoxin.

### Ionizing radiation

Cells and mice were irradiated at room temperature in ambient air using the Faxitron RX-650 irradiator (Faxitron X-ray, Wheeling, IL) at the indicated doses described in each experiment.

### Cell viability assay

The 3-(4,5-dimethylthiazol-2-yl)-2,5-diphenyl tetrazolium bromide (MTT) assay was used to determine the effects of CDT on the growth of PCa cells [54]. Briefly, PCa cells were treated with various concentrations of CDT and different periods. Relative cell number was quantified by a spectrophotometer (BioRad, Hercules, CA) at the wavelength of 570nm.

### Clonogenic survival assay

The surviving fraction (SF) of each treatment in cells was assessed using our previous report with a slight modification [10]. Briefly, cells were plated for 3 h to allow cell attachment followed by increasing doses of IR alone (2–6 Gy), CDT alone (1–10 nM), or CDT combined with IR. After 10-day incubation, the colonies were fixed and stained with 4% formaldehyde in PBS containing 0.05% crystal violet. The number of surviving colonies (defined as a colony with > 50 cells) was counted as (mean colony counts) / (cells inoculated) / (plating efficiency) and the plating efficiency was defined as (mean colony counts) / (cells inoculated for un-irradiated controls). The data are presented as the mean ± SEM of three independent experiments. The curve S = e^−(αD+ßD2)^ was fitted to the experimental data using a least squares fitting algorithm. Linear Quadratic (LQ) analysis was subjected for radiation surviving curve and calculated with the program Sigma Plot 11.0 (Systat Software, San Jose, CA). The determination of dose enhancement ratio (DER) was performed as described previously [49]. The radiation DER was calculated as the dose (Gy) for radiation alone divided by the dose for radiation combined with CDT (normalized for CDT toxicity).

### Cell cycle analysis

LAPC4 cells were treated with CDT (50 nM), IR (2 Gy), or CDT combined with IR. Cells were then incubated at 37°C for 0.5, 2, 8, 12, 24, and 48 h. The treated cells were harvested and fixed with ice-cold 70% ethanol for 1 h, and stained with 20 μg/ml propidium iodide (Sigma-Aldrich) containing 1 mg/ml RNase (Sigma-Aldrich) for 1 h. The stained cells were determined by an FACScalibur flow cytometer (Becton-Dickinson, San Jose, CA) and the data were analyzed using Cell Quest software WinMDI (Verity Software House, Topsham, Me).

### Western blot assay

Cells treated with CDT, IR, or CDT combined with IR for 24 h were harvested and cell lysate was prepared. The samples were then resolved by 6-12% SDS-PAGE and transferred onto polyvinylidene difluoride membranes (Millipore). Membranes were probed with primary antibodies as indicated and then incubated with horseradish peroxidase–conjugated secondary antibody (Millipore). The proteins of interest were detected using the ECL Western Blotting Detection Reagents (GE Healthcare, Piscataway, NJ) and visualized using X-ray film (Kodak, Rochester, NY). The signal intensity of each protein was quantified with the Image J software (National Institute of Health, Bethesda, MD).

### Immunofluorescence staining

Cells (1 × 10^6^ cells/well) were seeded on 13-mm glass coverslip in 6-well plates. After treatment, cells were washed and fixed with 1% paraformaldehyde (Sigma-Aldrich) and permeabilized with 0.1% Triton X-100 for 30 min at room temperature. The samples were blocked with 1% BSA for 1h and incubated with phospho-histone-γ-H2AX antibody (Ser139) (Millipore) and p53-binding protein 1 (53BP1) antibody (Santa Cruz). Samples were washed 3 times for 5 min in PBS, and then incubated with Alexa Fluor 488-conjugated anti-mouse antibody and Alexa Fluor 594-conjugated anti-rabbit antibody (Invitrogen) for 1 h. Nuclei were counterstained with DAPI (0.2 μg/ml) for 10 min. The stained cells were then analyzed under a fluorescence microscope (Carl Zeiss, Göttingen, Germany) with a 63× objective (oil immersion, aperture 1.3). Fifty nuclei from each experiment were counted and evaluated. All samples were examined in three independent experiments.

### Animal study

Male BALB/c nude mice (BALB/cAnN.Cg-*Foxnl^nu^*/CrlNarl, 5-weeks old; National Laboratory Animal Center) were used in this study. A suspension of LAPC4 shDAB2IP (3 × 10^6^) cells mixed with 50% Matrigel (BD Biosciences, Bedford, MA) in 0.1 ml was injected subcutaneously into the right posterior flanks of the mice. Animals were divided into four groups (9 animals/group), including untreated control, CDT alone (2.5 mg/kg), radiation alone and combined treatment of CDT and irradiation. CDT was administered intratumorly 6 h before radiation in the combination treatment group. Tumors were treated with a total dose of 12 Gy delivered in 3 fractions using the Faxitron RX-650 irradiator (Faxitron X-ray, Wheeling, IL) on day 0, 4 and 8. Tumors were measured 3 times per week using a Vernier caliper. Results were evaluated with the formula: volume = 0.5236 × length × width × height [55].

### Immunohistochemistry (IHC) analysis

Tissue specimens from mice were formalin-fixed then subjected to hematoxylin-eosin (H&E) or IHC staining. Briefly, the tissue sections were de-waxed and rehydrated. After blocking with 3% BSA, rabbit monoclonal antibodies against cleaved PARP (Cell Signaling) and Ki-67 (Thermo Fisher Scientific) were added to the tissue sections for 24 h at 4°C. After washing, the samples were probed with peroxidase-labeled goat anti-rabbit secondary antibody (Epitomics, Burlinggame, CA) and detected with an ABC kit (Vector Laboratories, Burlingame, CA).

DNA fragmentation was detected by terminal deoxynucleotidyl transferase-meditated dUTP-fluorescein nick end-labeling (TUNEL) with the fragmented DNA detection kit (Millipore, Temecula, CA) by following the manufacture's instruction. The prepared tissues were treated with permeabilization reagent (0.1% Triton X-100 in 0.1% sodium citrate) on ice for 2 min followed by incubation with the TUNEL reaction mixture at 37°C for 60 min. The stained tissues were analyzed under a fluorescence microscope (Carl Zeiss, Göttingen, Germany).

### Comet assay

Three cell lines (LAPC4, PC-3, and PZ-HPV-7) transfected with either shVector or shDAB2IP were left untreated or treated with 200 nM CDT for 24 h. The cells were then collected and prepared using the Comet Assay Kit (Trevigen, Gaithersburg, MD, USA). The nuclei were stained with propidium iodide (Sigma-Aldrich Corporation, St. Louis, MO, USA). The comets were imaged via fluorescence microscopy (Carl Zeiss, Göttingen, Germany). The tail moments (tail DNA% × tail length) were quantified from 50 randomly selected cells.

### Statistical analysis

Statistical analyses for the data between two groups were determined using Student *t-*test. Statistics analysis comparisons of more than two groups were evaluated using two-way analysis of variance (ANOVA). *P* < 0.05 was considered statistically significant. The statistical software was the SPSS program (version 12.0 for windows, SPSS Inc., Chicago, IL).

## SUPPLEMENTARY MATERIAL AND FIGURES



## References

[1] Sim HG, Cheng CW (2005). Changing demography of prostate cancer in Asia. Eur J Cancer.

[2] Dearnaley DP, Khoo VS, Norman AR, Meyer L, Nahum A, Tait D, Yarnold J, Horwich A (1999). Comparison of radiation side-effects of conformal and conventional radiotherapy in prostate cancer: a randomised trial. Lancet.

[3] Liu HH, Tsai YS, Lai CL, Tang CH, Lai CH, Wu HC, Hsieh JT, Yang CR (2013). Evolving personalized therapy for castration-resistant prostate cancer. BioMedicine.

[4] Hummerich J, Werle-Schneider G, Popanda O, Celebi O, Chang-Claude J, Kropp S, Mayer C, Debus J, Bartsch H, Schmezer P (2006). Constitutive mRNA expression of DNA repair-related genes as a biomarker for clinical radio-resistance: A pilot study in prostate cancer patients receiving radiotherapy. Int J Radiat Biol.

[5] Trock BJ, Han M, Freedland SJ, Humphreys EB, DeWeese TL, Partin AW, Walsh PC (2008). Prostate cancer-specific survival following salvage radiotherapy vs observation in men with biochemical recurrence after radical prostatectomy. JAMA.

[6] Wright JL, Dalkin BL, True LD, Ellis WJ, Stanford JL, Lange PH, Lin DW (2010). Positive surgical margins at radical prostatectomy predict prostate cancer specific mortality. J Urol.

[7] Zhou J, Scholes J, Hsieh JT (2003). Characterization of a novel negative regulator (DOC-2/DAB2) of c-Src in normal prostatic epithelium and cancer. J Biol Chem.

[8] Xie D, Gore C, Zhou J, Pong RC, Zhang H, Yu L, Vessella RL, Min W, Hsieh JT (2009). DAB2IP coordinates both PI3K-Akt and ASK1 pathways for cell survival and apoptosis. Proc Natl Acad Sci U S A.

[9] Xie D, Gore C, Liu J, Pong RC, Mason R, Hao G, Long M, Kabbani W, Yu L, Zhang H, Chen H, Sun X, Boothman DA, Min W, Hsieh JT (2010). Role of DAB2IP in modulating epithelial-to-mesenchymal transition and prostate cancer metastasis. Proc Natl Acad Sci U S A.

[10] Kong Z, Xie D, Boike T, Raghavan P, Burma S, Chen DJ, Habib AA, Chakraborty A, Hsieh JT, Saha D (2010). Downregulation of human DAB2IP gene expression in prostate cancer cells results in resistance to ionizing radiation. Cancer Res.

[11] Lara-Tejero M, Galan JE (2000). A bacterial toxin that controls cell cycle progression as a deoxyribonuclease I-like protein. Science.

[12] Lara-Tejero M, Galan JE (2002). Cytolethal distending toxin: limited damage as a strategy to modulate cellular functions. Trends Microbiol.

[13] Pickett CL, Pesci EC, Cottle DL, Russell G, Erdem AN, Zeytin H (1996). Prevalence of cytolethal distending toxin production in Campylobacter jejuni and relatedness of Campylobacter sp. cdtB gene. Infect Immun.

[14] Nesic D, Hsu Y, Stebbins CE (2004). Assembly and function of a bacterial genotoxin. Nature.

[15] Boesze-Battaglia K, Brown A, Walker L, Besack D, Zekavat A, Wrenn S, Krummenacher C, Shenker BJ (2009). Cytolethal distending toxin-induced cell cycle arrest of lymphocytes is dependent upon recognition and binding to cholesterol. J Biol Chem.

[16] Boesze-Battaglia K, Besack D, McKay T, Zekavat A, Otis L, Jordan-Sciutto K, Shenker BJ (2006). Cholesterol-rich membrane microdomains mediate cell cycle arrest induced by Actinobacillus actinomycetemcomitans cytolethal-distending toxin. Cell Microbiol.

[17] Lin CD, Lai CK, Lin YH, Hsieh JT, Sing YT, Chang YC, Chen KC, Wang WC, Su HL, Lai CH (2011). Cholesterol depletion reduces entry of Campylobacter jejuni cytolethal distending toxin and attenuates intoxication of host cells. Infect Immun.

[18] Lai CH, Lai CK, Lin YJ, Hung CL, Chu CH, Feng CL, Chang CS, Su HL (2013). Characterization of Putative Cholesterol Recognition/Interaction Amino Acid Consensus-Like Motif of Campylobacter jejuni Cytolethal Distending Toxin C. PLoS One.

[19] Yamamoto K, Tominaga K, Sukedai M, Okinaga T, Iwanaga K, Nishihara T, Fukuda J (2004). Delivery of cytolethal distending toxin B induces cell cycle arrest and apoptosis in gingival squamous cell carcinoma in vitro. Eur J Oral Sci.

[20] Iwanaga K, Tominaga K, Yamamoto K, Habu M, Maeda H, Akifusa S, Tsujisawa T, Okinaga T, Fukuda J, Nishihara T (2007). Local delivery system of cytotoxic agents to tumors by focused sonoporation. Cancer Gene Ther.

[21] Damek-Poprawa M, Volgina A, Korostoff J, Sollecito TP, Brose MS, O'Malley BW, Akintoye SO, DiRienzo JM (2011). Targeted inhibition of CD133+ cells in oral cancer cell lines. J Dent Res.

[22] Comayras C, Tasca C, Peres SY, Ducommun B, Oswald E, De Rycke J (1997). Escherichia coli cytolethal distending toxin blocks the HeLa cell cycle at the G2/M transition by preventing cdc2 protein kinase dephosphorylation and activation. Infect Immun.

[23] Guerra L, Albihn A, Tronnersjo S, Yan Q, Guidi R, Stenerlow B, Sterzenbach T, Josenhans C, Fox JG, Schauer DB, Thelestam M, Larsson LG, Henriksson M, Frisan T (2010). Myc is required for activation of the ATM-dependent checkpoints in response to DNA damage. PLoS One.

[24] Ohara M, Oswald E, Sugai M (2004). Cytolethal distending toxin: a bacterial bullet targeted to nucleus. J Biochem (Tokyo).

[25] Ishitoya S, Kurazono H, Nishiyama H, Nakamura E, Kamoto T, Habuchi T, Terai A, Ogawa O, Yamamoto S (2004). Verotoxin induces rapid elimination of human renal tumor xenografts in SCID mice. J Urol.

[26] Frankel AE, Rossi P, Kuzel TM, Foss F (2002). Diphtheria fusion protein therapy of chemoresistant malignancies. Curr Cancer Drug Targets.

[27] Michl P, Buchholz M, Rolke M, Kunsch S, Lohr M, McClane B, Tsukita S, Leder G, Adler G, Gress TM (2001). Claudin-4: a new target for pancreatic cancer treatment using Clostridium perfringens enterotoxin. Gastroenterology.

[28] Wu K, Xie D, Zou Y, Zhang T, Pong RC, Xiao G, Fazli L, Gleave M, He D, Boothman DA, Hsieh JT (2013). The Mechanism of DAB2IP in Chemoresistance of Prostate Cancer Cells. Clin Cancer Res.

[29] Min J, Zaslavsky A, Fedele G, McLaughlin SK, Reczek EE, De Raedt T, Guney I, Strochlic DE, Macconaill LE, Beroukhim R, Bronson RT, Ryeom S, Hahn WC, Loda M, Cichowski K (2010). An oncogene-tumor suppressor cascade drives metastatic prostate cancer by coordinately activating Ras and nuclear factor-kappaB. Nat Med.

[30] Duggan D, Zheng SL, Knowlton M, Benitez D, Dimitrov L, Wiklund F, Robbins C, Isaacs SD, Cheng Y, Li G, Sun J, Chang BL, Marovich L, Wiley KE, Balter K, Stattin P, Adami HO, Gielzak M, Yan G, Sauvageot J, Liu W, Kim JW, Bleecker ER, Meyers DA, Trock BJ, Partin AW, Walsh PC, Isaacs WB, Gronberg H, Xu J, Carpten JD (2007). Two genome-wide association studies of aggressive prostate cancer implicate putative prostate tumor suppressor gene DAB2IP. J Natl Cancer Inst.

[31] McGuire BB, Helfand BT, Kundu S, Hu Q, Banks JA, Cooper P, Catalona WJ (2011). Association of prostate cancer risk alleles with unfavourable pathological characteristics in potential candidates for active surveillance. BJU Int.

[32] Helfand BT, Hu Q, Loeb S, McVary KT, Catalona WJ (2012). Genetic sequence variants are associated with severity of lower urinary tract symptoms and prostate cancer susceptibility. J Urol.

[33] Lange EM, Salinas CA, Zuhlke KA, Ray AM, Wang Y, Lu Y, Ho LA, Luo J, Cooney KA (2012). Early onset prostate cancer has a significant genetic component. Prostate.

[34] Yu L, Tumati V, Tseng SF, Hsu FM, Kim DN, Hong D, Hsieh JT, Jacobs C, Kapur P, Saha D (2012). DAB2IP regulates autophagy in prostate cancer in response to combined treatment of radiation and a DNA-PKcs inhibitor. Neoplasia.

[35] Pickett CL, Whitehouse CA (1999). The cytolethal distending toxin family. Trends Microbiol.

[36] Bartek J, Bartkova J, Lukas J (2007). DNA damage signalling guards against activated oncogenes and tumour progression. Oncogene.

[37] Su TT (2006). Cellular responses to DNA damage: one signal, multiple choices. Annu Rev Genet.

[38] Grandori C, Cowley SM, James LP, Eisenman RN (2000). The Myc/Max/Mad network and the transcriptional control of cell behavior. Annu Rev Cell Dev Biol.

[39] Fernandez-Capetillo O, Celeste A, Nussenzweig A (2003). Focusing on foci: H2AX and the recruitment of DNA-damage response factors. Cell Cycle.

[40] Leontieva OV, Lenzo F, Demidenko ZN, Blagosklonny MV (2012). Hyper-mitogenic drive coexists with mitotic incompetence in senescent cells. Cell Cycle.

[41] Darzynkiewicz Z (2009). When senescence masquerades as DNA damage: Is DNA replication stress the culprit?. Cell Cycle.

[42] Pospelova TV, Demidenko ZN, Bukreeva EI, Pospelov VA, Gudkov AV, Blagosklonny MV (2009). Pseudo-DNA damage response in senescent cells. Cell Cycle.

[43] Angele S, Falconer A, Foster CS, Taniere P, Eeles RA, Hall J (2004). ATM protein overexpression in prostate tumors: possible role in telomere maintenance. Am J Clin Pathol.

[44] Bonner WM, Redon CE, Dickey JS, Nakamura AJ, Sedelnikova OA, Solier S, Pommier Y (2008). GammaH2AX and cancer. Nat Rev Cancer.

[45] Alaoui-El-Azher M, Mans JJ, Baker HV, Chen C, Progulske-Fox A, Lamont RJ, Handfield M (2010). Role of the ATM-checkpoint kinase 2 pathway in CDT-mediated apoptosis of gingival epithelial cells. PLoS One.

[46] Ponnurangam S, Mammen JM, Ramalingam S, He Z, Zhang Y, Umar S, Subramaniam D, Anant S (2012). Honokiol in combination with radiation targets notch signaling to inhibit colon cancer stem cells. Mol Cancer Ther.

[47] Hennequin C, Giocanti N, Favaudon V (1996). Interaction of ionizing radiation with paclitaxel (Taxol) and docetaxel (Taxotere) in HeLa and SQ20B cells. Cancer Res.

[48] Azria D, Jacot W, Prost P, Culine S, Ychou M, Lemanski C, Dubois JB (2002). Gemcitabine and ionizing radiations: radiosensitization or radio-chemotherapy combination. Bull Cancer.

[49] Kong Z, Raghavan P, Xie D, Boike T, Burma S, Chen D, Chakraborty A, Hsieh JT, Saha D (2010). Epothilone B confers radiation dose enhancement in DAB2IP gene knock-down radioresistant prostate cancer cells. Int J Radiat Oncol Biol Phys.

[50] Rowinsky EK, Donehower RC (1995). Paclitaxel (taxol). N Engl J Med.

[51] Anderson P, Aguilera D, Pearson M, Woo S (2008). Outpatient chemotherapy plus radiotherapy in sarcomas: improving cancer control with radiosensitizing agents. Cancer Control.

[52] Wolf S, Barton D, Kottschade L, Grothey A, Loprinzi C (2008). Chemotherapy-induced peripheral neuropathy: prevention and treatment strategies. Eur J Cancer.

[53] Lai CK, Lu YL, Hsieh JT, Tsai SC, Feng CL, Tsai YS, Tsai PC, Su HL, Lin YH, Lai CH (2013). Development of chitosan/heparin nanoparticle-encapsulated cytolethal distending toxin for gastric cancer therapy. Nanomedicine (Lond).

[54] Lu DY, Tang CH, Chang CH, Maa MC, Fang SH, Hsu YM, Lin YH, Lin CJ, Lee WC, Lin HJ, Lee CH, Lai CH (2012). Helicobacter pylori attenuates lipopolysaccharide-induced nitric oxide production by murine macrophages. Innate Immun.

[55] Pong RC, Roark R, Ou JY, Fan J, Stanfield J, Frenkel E, Sagalowsky A, Hsieh JT (2006). Mechanism of increased coxsackie and adenovirus receptor gene expression and adenovirus uptake by phytoestrogen and histone deacetylase inhibitor in human bladder cancer cells and the potential clinical application. Cancer Res.

