# Supplementary material for: Sensitization of radio-resistant prostate cancer cells with a unique cytolethal distending toxin
Source: Oncotarget. 2014 Jun 26;5(14):5523–34. doi: 10.18632/oncotarget.2133 (PMC4170639; doi:10.18632/oncotarget.2133)
Supplement: Supplementary file 1 [file oncotarget-05-5523-s001.pdf]

Sensitization of radio-resistant prostate cancer cells with a unique cytolethal distending toxin

Supplementary Material

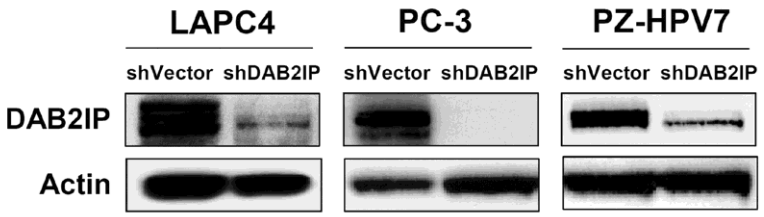

Supplementary Figure S1: Western blot analysis of DAB2IP expression in shVector (control) and shDAB2IP (i.e., DAB2IP-deficient) PCa cells.

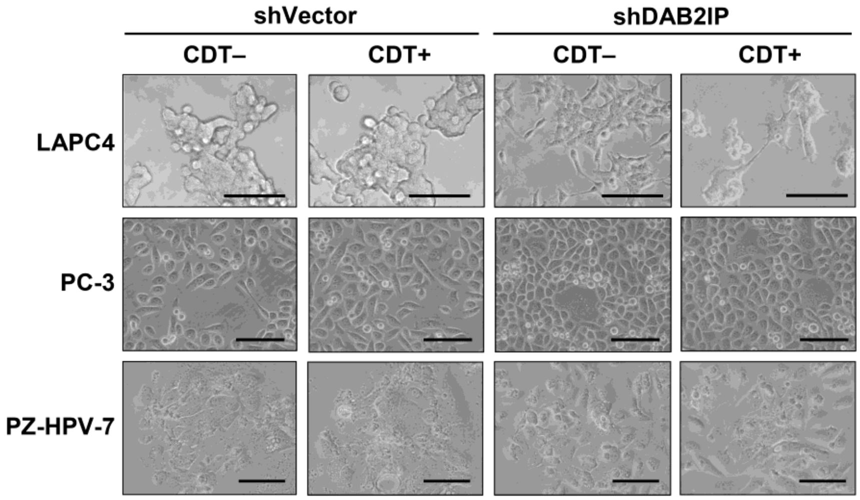

Supplementary Figure S2: Cell distending activity of CDT in DAB2IP-deficient PCa cells. shVector or shDAB2IP PCa cells were untreated (CDT-) or treated (CDT+) with 200 nM CDT holotoxin at 37°C for 24 h. The cells were then examined under an inverted optical microscope to assess the effects of CDT intoxication (scale bar, 50 µm).

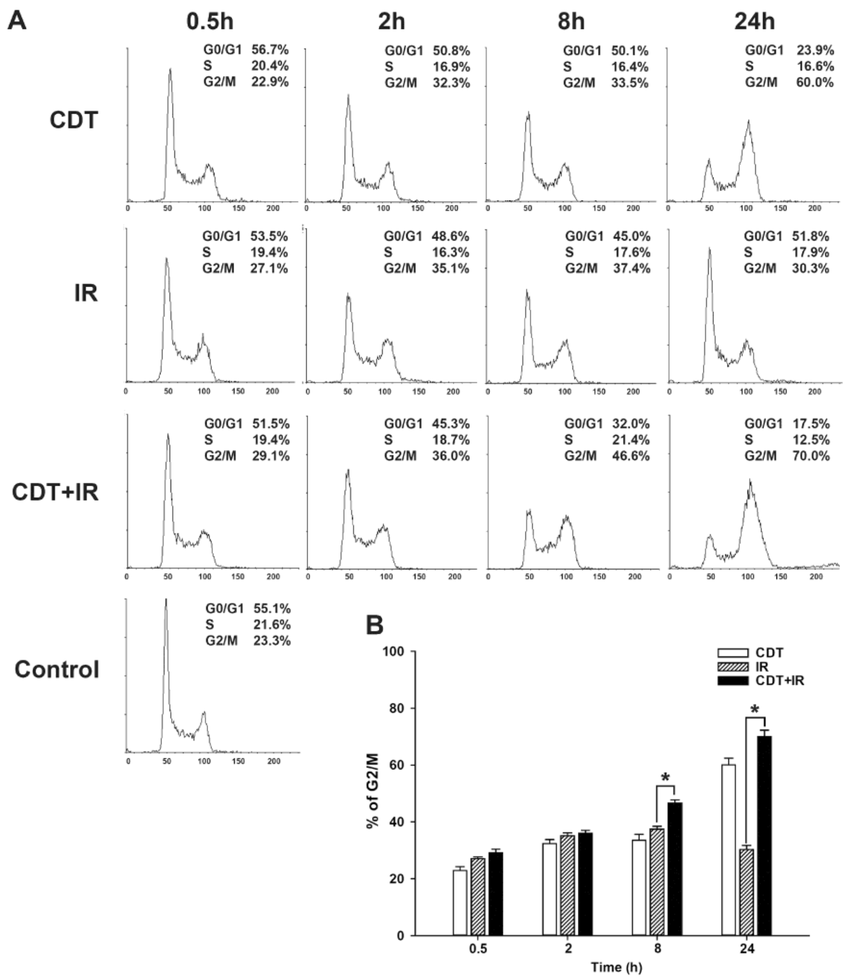

Supplementary Figure S3: Overexpression of c-Myc increases CDT-induced cell death. (A) shVector cells were transfected with c-Myc for 24 h, and the c-Myc protein level was analyzed by western blot analysis. (B) Cells were then exposed to 200 nM CDT for an additional 24 h, and the cytotoxicity of CDT in shVector cells was analyzed using the MTT assay. The asterisk (*) indicates statistical significance ($P < 0.05$) determined by Student's *t*-test.

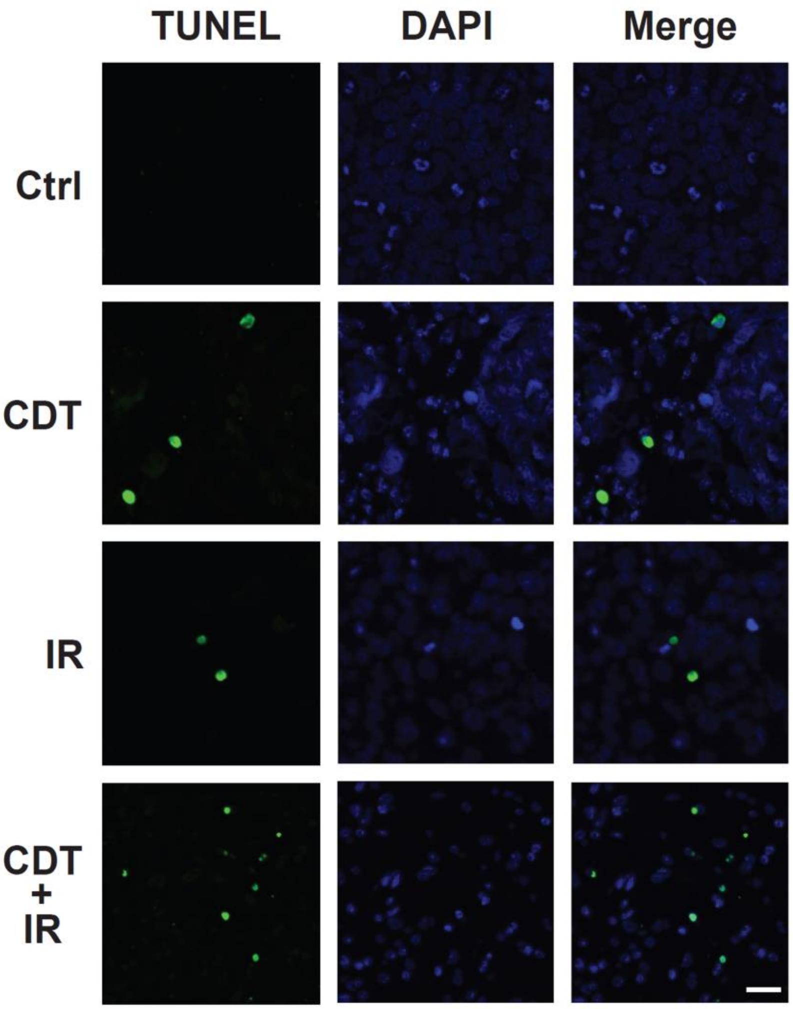

Supplementary Figure S4: Apoptosis contributes to tumor cell death in mice treated with a combination of CDT and IR. Representative PCa xenograft samples from each treatment group (vehicle control, CDT alone, IR alone, and CDT combined with IR) were assessed by the TUNEL assay. Cells stained with TUNEL (green) and DAPI (blue) were observed under a fluorescence microscope (scale bar, 10 μm).

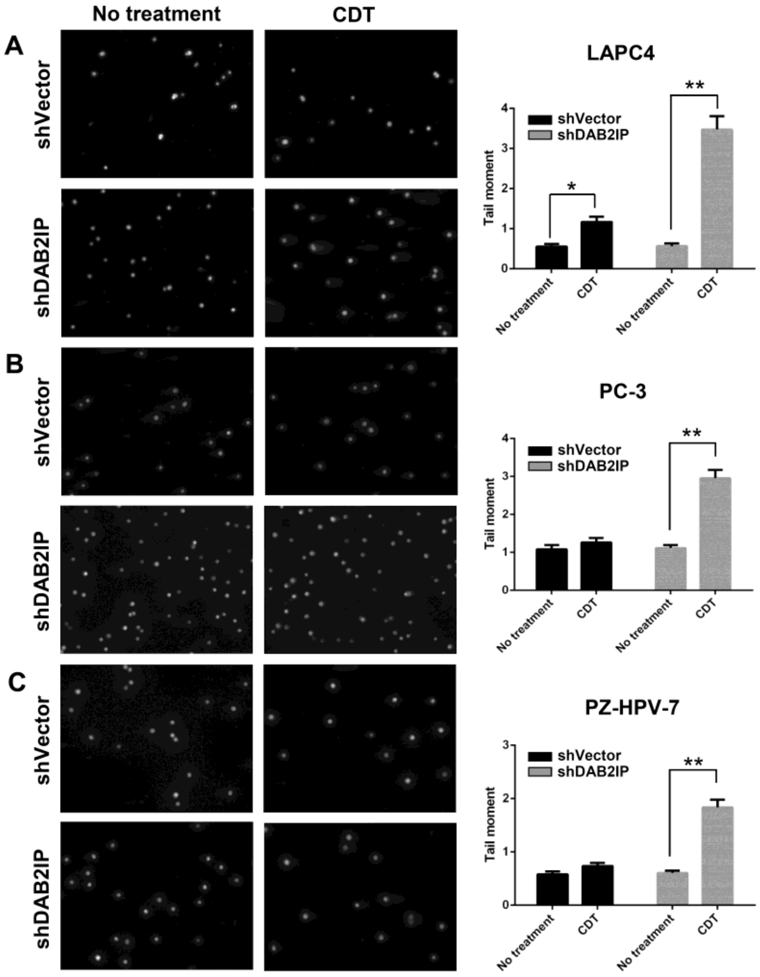

Supplementary Figure S5: Detection of CDT-induced DNA damage by comet assay. (A) LAPC4, (B) PC3, and (C) PZ-HPV-7 cells transfected with either shVector or shDAB2IP were either left untreated or treated with CDT (200 nM) for 24 h. The cells were then electrophoresed and stained as described in the Methods section. The labeled DNA was visualized under a fluorescence microscope. The tail moment was quantified for each cell; these data are presented as histograms in the right-hand panels. Statistical significance was evaluated using Student's *t*-test (*, $P < 0.05$; **, $P < 0.01$).
